# Pervaporation and Gas Separation Properties of High-Molecular Ladder-like Polyphenylsilsesquioxanes

**DOI:** 10.3390/polym15153277

**Published:** 2023-08-02

**Authors:** Tatiana S. Anokhina, Tatyana O. Ershova, Anton A. Anisimov, Maxim N. Temnikov, Evgenia A. Grushevenko, Ilya L. Borisov, Alexey V. Volkov, Aziz M. Muzafarov

**Affiliations:** 1 V. Topchiev Institute of Petrochemical Synthesis RAS, 119991 Moscow, Russia; evgrushevenko@ips.ac.ru (E.A.G.); boril@ips.ac.ru (I.L.B.); avolkov@ips.ac.ru (A.V.V.); 2 N. Nesmeyanov Institute of Organoelement Compounds RAS, 119334 Moscow, Russia; ershovatatyana_2995@mail.ru (T.O.E.); aziz@ispm.ru (A.M.M.); 3 The Faculty of Natural Sciences, Tula State Lev Tolstoy Pedagogical University, 300026 Tula, Russia; 4 Moscow Institute of Physics and Technology, Faculty of Electronics, Photonics and Molecular Physics, National Research University, 141700 Dolgoprudny, Russia; 5 Enikolopov Institute of Synthetic Polymeric Materials RAS, 117393 Moscow, Russia

**Keywords:** polypheylsilsesquioxanes, membranes, pervaporation, gas separation

## Abstract

This paper presents the results of studies on the pervaporation properties (for benzene/hexane mixtures) and gas permeability (for He, H_2_, N_2_, O_2_, CO_2_, CH_4_, C_2_H_6_, and C_4_H_10_) of ladder-like polyphenylsesquioxanes (L-PPSQ) with improved physical and chemical properties. These polymers were obtained by condensation of *cis*-tetraphenylcyclotetrasiloxanetetraol in ammonia medium. The structure of L-PPSQ was fully confirmed by a combination of physicochemical analysis methods: ^1^H, ^29^Si NMR, IR spectroscopy, HPLC, powder XRD, and viscometry in solution. For the first time, a high molecular weight of the polymer (Mn = 238 kDa, Mw = 540 kDa) was achieved, which determines its improved mechanical properties and high potential for use in membrane separation. Using TGA and mechanical analysis methods, it was found that this polymer has high thermal (T_d_^5%^ = 537 °C) and thermal-oxidative stability (T_d_^5%^ = 587 °C) and good mechanical properties (Young’s module (E) = 1700 MPa, ultimate tensile stress (σ) = 44 MPa, elongation at break (ε) = 6%), which is important for making membranes workable under various conditions. The polymer showed a high separation factor for a mixture of 10% wt. benzene in n-hexane (126) at a benzene flow of 33 g/(m^2^h).

## 1. Introduction

Membrane separation processes are promising for CO_2_ extraction [1] and for the separation of aromatic/aliphatic hydrocarbons [2,3]. Membrane technologies allow separation costs to be reduced significantly owing to their compactness, modularity, higher separation efficiency, and lower energy consumption compared to conventional processes. High permeability, selectivity, and stability at elevated temperatures are the key properties of membrane materials in the development of membranes based on them [4].

Polymer membranes based on glassy polymers currently dominate the market because of their relatively low cost, ease of production, and scalability of the molding process [5]. Though membrane polymers can work at elevated temperatures, only polytetrafluoroethylene (PTFE) and polysulfones are mechanically and hydrolytically resistant to high-temperature wet gases. The creation of membranes based on PTFE for the separation of gases and organic solvents does not appear possible due to its insolubility in organic solvents, acids, and alkalis [6]. The operating temperatures of polysulfones do not exceed 170–180 °C [7,8,9].

Polysiloxane polymers became widely popular as membrane materials [10,11,12], primarily due to their high permeability, stability of transport properties, and chemical and thermal stability. Polysiloxanes are stable due to the strength of siloxane bonds. The unique flexibility of the polysiloxane chain along with the weak intra- and intermolecular interactions provide high values of free volume and segmental mobility of the polymer [13]. In processes where the transport occurs by the dissolution–diffusion mechanism, siloxanes are advantageous in the separation of well-sorbed components. In combination with low diffusion selectivity, this capability of membranes is relevant for the separation of large organic molecules from liquid [11] or gaseous media [13,14,15].

The properties of siloxane polymers are mainly determined by the structure of the main chain and the type of organic substituent at the silicon atom. Membranes based on linear (polydimethylsiloxane (PDMS) and (polymethyloctylsiloxane (POMS)) and substituted polysiloxanes are used in most processes where continuous selective layer membranes are used: gas separation [16], vapor separation [17], pervaporation [18], nanofiltration of organic media [19], perstraction [20], gas–liquid membrane contactors [21], and even electrodialysis [22].

Polyphenylsilsesquioxanes (PPSQ) are a class of organosilicon compounds that possess a set of unique physical and chemical properties, such as high thermal and radiation stability, good mechanical properties, a high refractive index, and the ability to be dissolved in a wide range of organic solvents. Due to the combination of these properties, PPSQ are used in various fields of science and technology. Polyphenylsilsesquioxanes are often incorporated into composites [23,24] and copolymers [25] in order to impart new properties to materials. PPSQ are also widely applied as protective, hydrophobic, and heat-resistant coatings [26], and their good optical properties make them ideal materials for optoelectronics [27,28,29].

The structure of polyphenylsilsesquioxanes directly depends on the method used to produce them. In general, PPSQ are obtained by hydrolytic polycondensation of a trifunctional monomer, phenyltrichloro- or alkoxysilane. Polyhedral, superbranched, statistical, or ladder-like structures can be obtained by varying the synthesis conditions [30]. The properties of each structural form differ dramatically [31,32]. 

Among all the structural types of PPSQ, ladder-like polyphenylsilsesquioxanes (L-PPSQ) are the most interesting and well studied. L-PPSQ were first obtained in 1960 by Brown et al. by high-temperature polymerization of phenyltrichlorosilane hydrolysis products [33]. This is a classical method whose main advantage is that polymers with a high molecular weight (~10^6^ Da) are obtained. Such high-molecular-weight L-PPSQ have high thermal and thermal-oxidative stability (over 500 °C) along with the ability to form strong transparent films. The works of K.A. Andrianov and V.N. Tsvetkov, who published numerous papers dealing with the development of L-PPSQ synthesis methods and their properties, made a great contribution to the study of L-PPSQ [34,35,36,37]. However, the drawbacks of the methods that they presented, such as the need for multistage processes, the use of large amounts of solvents and catalysts, and the drastic conditions required for the reactions, restrained their wide practical application. 

In recent years, a number of scientific teams have suggested alternative methods for synthesizing polymers of this class [38,39,40]. However, despite the milder process conditions, they still involve multiple stages, require the use of solvents and catalysts, and, most importantly, cannot provide polymers with a high molecular weight. A significant drawback of the above methods is that the resulting L-PPSQ are brittle, which further restricts the potential of their use in modern materials science. Thus, at present, the search for a new and efficient method for the synthesis of high-molecular-weight L-PPSQ is undoubtedly a pressing task.

Previously, the condensation of phenyl-containing silanols in ammonia medium using model compounds with 1, 2, or 3 silanol groups (triphenylsilanol, diphenylsilanediol, tetraphenyldisiloxanediol, and phenylsilanetriol) was studied at Institute of the Organoelement of the Russian Academy of Sciences. It was found that this method could provide high yields of phenyl-containing siloxanes with various structures [41]. Later, a simple and highly efficient method for the synthesis of ladder-like polyphenylsilsesquioxanes with controlled molecular weights in ammonia medium was developed and optimized [42]. The main difference between the L-PPSQ obtained in ammonia medium and the L-PPSQ obtained by other methods is that the former are less brittle, which, combined with their good mechanical and thermal characteristics, makes them promising objects for use in materials science.

The purpose of this work was to obtain and study the gas separation and pervaporation properties of L-PPSQ with improved physical and chemical characteristics.

## 2. Experimental

### 2.1. Materials

Solvents were purified according to the reported procedures [43]. Toluene was distilled from calcium hydride under argon. Sodium hydroxide, pyridine, chlorotrimethylsilane, and HCl were purchased from Aldrich. All-*cis*-tetraphenylcyclotetrasiloxanol was obtained by our method, published previously [44].

Anhydrous ammonia was purchased from Spectra Gases Inc., Gatineau Quebec, Canada.

The solubility of the polymer was studied in benzene (“chemically pure” grade 99.8%, ChimMed, Moscow, Russia), n-methylpyrrolidone (ReagentPlus^®^ 99%, Sigma-Aldrich, St. Louis, MO, USA), dimethylacetamide (99%, DuPont, Wilmington, DE, USA), hexane (“chemically pure” grade 99.0%, ECOS-1, Moscow, Russia), acetone (“pure for analysis” grade, 99.75%, Komponent-Reaktiv, Moscow, Russia), and ethanol (96%, ChimMed, Moscow, Russia).

### 2.2. Instrumentation

GPC analysis was performed on a Shimadzu chromatograph using a RID-20A refractometer as the detector, PSS SDV analytical 10^5^ Å columns (size 300 × 8 mm), and THF as the eluent.

NMR spectra were recorded on a Bruker Avance™ 600 spectrometer (Bruker Elemental GmbH, Karlsruhe, Germany) operating at 99.36 MHz for ^29^Si. The chemical shifts for ^29^Si were measured using TMS as the external standard.

IR spectra were obtained using a Bruker Tensor 37 FT-IR spectrometer ((Bruker Elemental GmbH, Karlsruhe, Germany).

Powder X-ray diffraction phase analysis was performed on a D8 Advance Bruker AXS (Bruker Elemental GmbH, Karlsruhe, Germany) diffractometer in Bragg-Brentano focusing geometry using CuKα radiation; the angular step was 0.02°, and the scan rate was 1–2 deg min^−1^. The samples were placed on flat holders. Powder patterns were processed with DIFFRACplus software Bruker AXS.

Thermogravimetric studies were performed using a Derivatograph-C instrument (MOM SZERVIZ KFT, Budapest, Hungary) under air or argon at a heating rate of 10 K min^–1^.

### 2.3. Synthetic Procedures

#### Synthesis of Ladder-like Polyphenylsilsesquioxane in Ammonia

**Figure 1:**
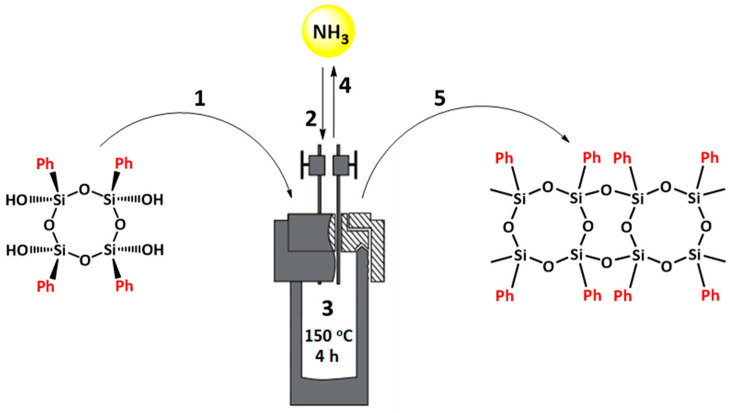
General scheme for L-PPSQ synthesis in ammonia medium.

The condensation of *cis*-tetraphenylcyclotetrasiloxanetraol (*cis*-tetrol) was performed in steel autoclaves (see Figure 1).

**Scheme 1:**
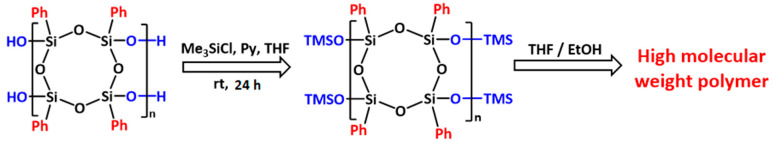
Blocking of residual silanol groups in L-PPSQ followed by reprecipitation of the resulting polymer.

*Cis*-tetrol (1 g) was loaded into an autoclave (1), which was then cooled to −50 °C, and ammonia was pumped in (2) using a gas flow regulator. The reaction was carried out at 150 °C for 4 h (3), after which ammonia was decompressed (4), thus isolating the target product (5). To determine the structure and properties of the resulting L-PPSQ, the residual silanol groups were blocked with chlorotrimethylsilane in the presence of pyridine, and then the resulting product was resuspended in the THF/EtOH system (Scheme 1).

It is important to note that L-PPSQ synthesized in ammonia has a high molecular weight, which cannot be achieved using other modern methods of synthesis of these polymers. The LPPSQ obtained in this work was investigated by uniaxial stretching and TGA methods. It can be seen from the data obtained that the synthesized polymer has high mechanical and thermal characteristics (see Table 1 and Supplementary Materials), which exceed the results published at the moment in the literature [45]. The main difference between L-PPSQ obtained in ammonia and the polymers obtained by other methods is their lower brittleness, which combined with good mechanical and thermal properties makes them promising objects for today’s materials science.

### 2.4. Study of L-PPSQ Solubility

The solubility of L-PPSQ was studied in a number of organic solvents: Chloroform, benzene, n-methylpyrrolidone, dimethylacetamide, hexane, acetone, ethanol, and a mixture of benzene and hexane in 10/90 and 30/70 ratios. The Hildebrand and Hansen solubility parameters were analyzed for each solvent and polymer.

### 2.5. Preparation of L-PPSQ Films

An L-PPSQ solution in chloroform (1 wt%) was used to make the films. Dense L-PPSQ films were fabricated by casting L-PPSQ solution in chloroform onto cellophane, followed by drying for 200 h under ambient conditions at room temperature. The initial film diameter was 7.5 cm. The thickness varied in the range of 30–35 μm. The film thickness was measured to within ±1 μm using a Mitutoyo^®^ 293 Digimatic QuickMike electron micrometer (Takatsu-ku, Kawasaki, Kanagawa, Japan).

### 2.6. Gas Transport Tests 

Single gas permeation measurements were carried out at a temperature of 30.0 ± 0.1 °C and at a feed pressure of 0.8 bar using a constant volume/variable pressure experimental setup (GKSS Time-Lag Machine) described elsewhere [46]. The permeability coefficient *P* expressed in Barrers (1 Barrer = 10^−10^ cm^3^ (STP) × cm × cm^−2^ × s^−1^ × cmHg^−1^) was estimated by linear extrapolation of experimental data to zero trans-membrane pressure. The diffusion coefficient *D* was estimated using the time-lag method as *D* = *l*^2^/6*θ*, where *l* is the membrane thickness and *θ* is the experimental time lag before the attainment of the steady-state permeation regime. The solubility coefficient *S* was indirectly estimated in terms of the solution–diffusion permeation model: *S* = *P*/*D*. The ideal selectivity of a pair of gases was calculated as the ratio of the permeabilities of the individual gases.

### 2.7. Study of Pervaporative Properties

The transport and separation properties of L-PPSQ membranes were studied by vacuum pervaporation for the separation of a benzene/hexane mixture containing from 5 to 15 wt. % benzene at 20 °C. The membrane thickness was 100 ± 5 μm.

**Figure 2:**
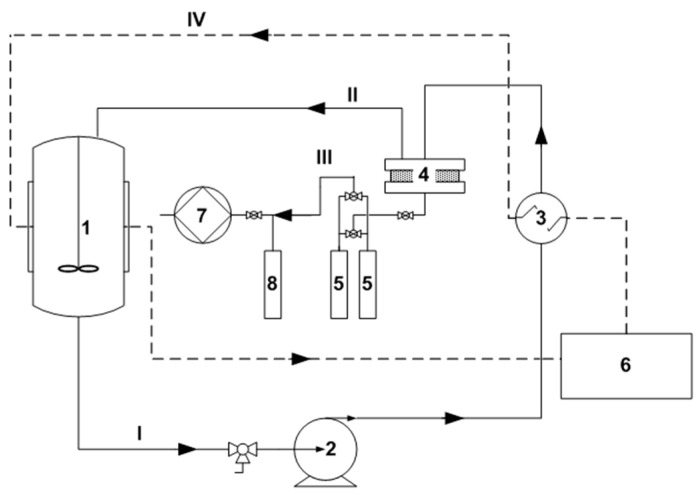
Schematic of vacuum permeate setup: 1—vessel with mixing device; 2—gear pump; 3—heat exchanger; 4—membrane module; 5—permeate collection traps placed in Dewar vessels with liquid nitrogen; 6—thermostat; 7—vacuum pump; 8—safety trap; I—starting mixture to be separated; II—retentate; III—permeate; IV—coolant.

Figure 2 shows a schematic diagram of the vacuum evaporation unit described elsewhere [47]. Using an MV-Z gear pump (Ismatec, Zurich, Switzerland) (2), the starting mixture to be separated (flow I) is fed from a thermostatically controlled 1 L container with a stirrer (1) through a heat exchanger (3) to a membrane module (4), and then back to the tank (1) by flow II. The effective membrane area in the membrane module is 13.5 cm^2^; the volume flow rate of the mixture being separated is 350 mL/min. The permeate passing through the membrane (flow III) in the vapor phase is condensed in glass traps (5) placed in Dewar flasks with liquid nitrogen (−196 °C). To achieve continuity of pervaporative separation, the traps are arranged in parallel and operate alternately throughout the experiment. A LOIP LT-100 liquid thermostat (St. Petersburg, Russia) (6) ensures a constant temperature of the mixture being separated with an accuracy of ±0.1 °C. The creation and maintenance of the difference in partial pressures of the mixture vapors were provided by evacuation of the submembrane space with an Ebara PDV-250 vacuum pump (Ota-ku, Tokyo, Japan) (7). The pressure in the diaphragm space was 0.2 mbar. The safety trap (8) prevents the permeate vapors from entering the vacuum pump.

The concentrations of the initial solution, the retentate, and the permeate were determined by gas chromatography on a Crystallux-4000M chromatograph (Yoshkar-ola, Russia) equipped with a flame ionization detector. Analysis was performed using the following parameters: Evaporation temperature of 200 °C, column temperature of 120 °C, and detector temperature of 150 °C. We used a Phenomenex Zebron ZB-FFAP capillary column (length 50 m, diameter 0.32 mm, phase thickness 0.50 µm) and the phase with the following composition: polymeric ester of 2-nitroterephthalic acid + polyethylene glycol.

The total permeate flow *J*, kg/(m^2^·h), was determined by the gravimetric method using the Equation (1):

(1) $$J=\frac{m}{S\cdot t}$$

where *m* is the total mass of the permeate that passed through a membrane with area *S* in time *t*.

The separation factor *β* was determined by Equation (2):

(2) $$\beta =\frac{y_{o}\cdot x_{w}}{y_{w}\cdot x_{o}}$$

where *x_o_* and *x_w_* are the mass fractions of the organic component and water in the mixture being separated, and *y_o_* and *y_w_* are the mass fractions of the organic component and water in the permeate.

The mass flows of the components in the permeate were determined as:

(3) $$J_{i}=J\cdot y_{o}$$

## 3. Results and Discussion

We used high-molecular-weight L-PPSQ obtained by condensation of *cis*-tetraphenylcyclotetrasiloxantetraol (*cis*-tetrol) in ammonia as the membrane material [42]. The main difference between L-PPSQ obtained in ammonia and the polymers obtained by other methods is that they have lower brittleness, which, combined with good mechanical (E = 1700 MPa, σ_p_ = 44 MPa, ε_p_ = 6%) and thermal characteristics (T_d_^5%^ above 500 °C in air and in argon) makes them promising objects for today’s materials science (see Supplementary Materials). 

An advantage of this method is that the ammonia used in *cis*-tetrol condensation acts both as a solvent and a catalyst in this process, which eliminates the need for additional reagents. Yet another important advantage of this approach is that ammonia is instantly removed from the reaction zone upon decompression. This makes it possible to obtain target products that require no additional purification. 

**Figure 3:**
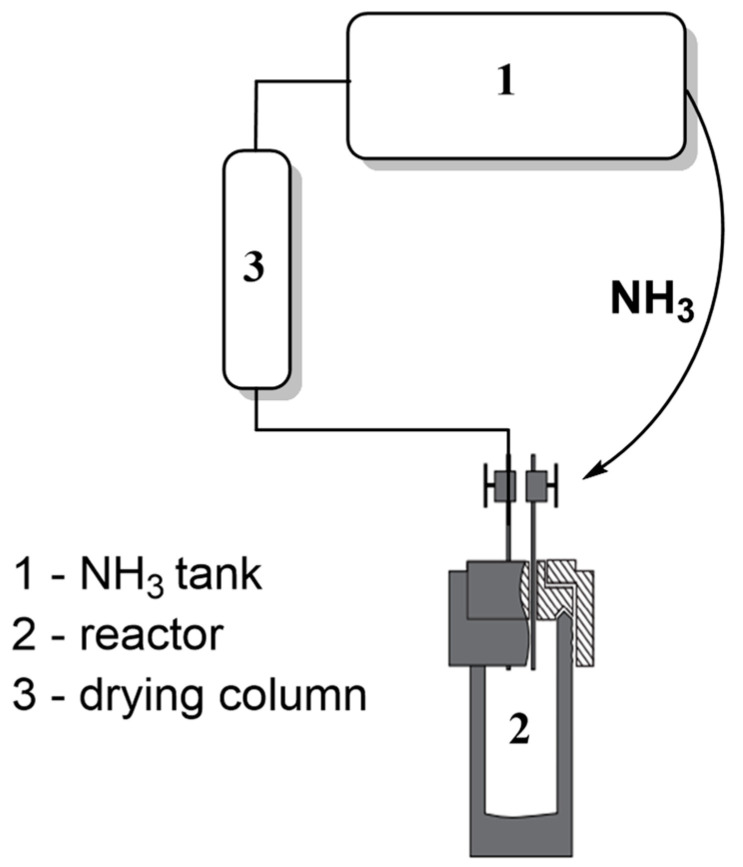
Schematic of the setup for ammonia recycling.

It is important to note that decompressed ammonia can be regenerated by means of a drying column (Figure 3), which allows it to be used repeatedly in further syntheses.

### 3.1. Ammonia Regeneration

It has been shown [42] that the presence of water in the reaction of *cis*-tetrol with ammonia results in products with low molecular weights. Water is also released in this process in the course of homocondensation. Accordingly, additional equipment must be used to dry the regenerated ammonia before it is reused. 

**Figure 4:**
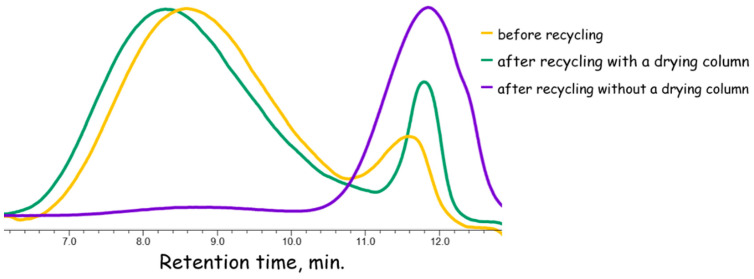
GPC curves of experiments with ammonia recycling.

After the reaction is performed in reactor 2, ammonia is pumped through the drying column 3 to the NH_3_ tank 1. After that, ammonia can be reused quantitatively in the next reaction. As can be seen from Figure 4, L-PPSQ obtained using regenerated ammonia has almost the same molecular weight characteristics as the polymer obtained in the reaction with the original ammonia. If ammonia is pumped to the next reaction without a drying column, then products with low molecular weights are obtained (see Figure 4). This important result of our study allows us to state that our suggested approach fully meets modern “green chemistry” requirements.

### 3.2. Study of L-PPSQ Solubility

To determine the sorption interaction of the polymer synthesized with various solvents, its solubility was studied in seven organic solvents of different nature (chloroform, benzene, n-methylpyrrolidone (NMP), dimethylacetamide (DMAA), hexane, acetone, and ethanol) and two organic mixtures (benzene/ethanol with different compositions) (Table 2). 

Table 2 shows that L-PPSQ is soluble in chloroform, benzene, and polar aprotic amide solvents: NMP and DMAA. L-PPSQ is insoluble in hexane, acetone, and ethanol. L-PPSQ is insoluble in a benzene/hexane mixture at a ratio of 10/90, but it swells strongly in the 30/70 mixture. This behavior is characteristic of PPSQ. Various groups of researchers noted the solubility of materials of this class in benzene, chloroform, and aprotic solvents (THF, DMF) and their insolubility in ketones, alcohols, simple ether, and saturated cyclic and aliphatic hydrocarbons [48]. The insolubility of L-PPSQ in a wide range of solvents allows us to expect that the properties of a membrane will be stable when the latter is operated in these media. Apparently, an undoubted advantage of L-PPSQ is that it is soluble in solvents with various viscosities, which would allow the development of membranes with good performance characteristics on its basis.

Based on the values of the long-range interaction parameter obtained, we can say that benzene and chloroform are the best solvents for L-PPSQ in the series studied. The interaction of L-PPSQ with NMP and DMAA solvents appears to occur at the boundary of the region of “good” solvents in terms of the long-range parameter value (MF: 10.6 MPa^1/2^; DMAA: 10.5 MPa^1/2^). In the 30 wt.% benzene/70 wt.% n-hexane mixture, L-PPSQ swells strongly but does not dissolve. Thus, it can be assumed that a long-distance parameter value above 10.5–10.6 delineates the region of “bad” solvents. 

### 3.3. Gas Transport Properties

The permeability (*P*) and diffusion (*D*) coefficients were measured for individual gases: He, H_2_, N_2_, O_2_, CO_2_, CH_4_, C_2_H_6_, and C_4_H_10_. The solubility coefficients (*S*) were calculated as the ratio of permeability coefficients to diffusion coefficients (Table 3). The ideal selectivity of permeability (*α_P_*), diffusion (*α_D_*), and solubility (*α_S_*) was defined as the ratio of the permeability, diffusion, and solubility coefficients for the corresponding gas pair (Table 4) according to Equation (4).

(4) $$\alpha_{P}=\frac{P_{i}}{P_{j}};\;\alpha_{D}=\frac{D_{i}}{D_{j}};\;\alpha_{S}=\frac{S_{i}}{S_{j}}$$

The largest diffusion coefficients are characteristic of gases with the smallest diffusion diameters, i.e., He and H_2_ (d_ef_ (He) = 2.10 Å and d_ef_ (H_2_) = 1.80 Å). The distribution of diffusion coefficients is characteristic of the silsesquioxane family. Due to the high diffusion of gases with small kinetic diameters, the L-PPSQ synthesized in this work has selective permeability for He and H_2_. Thus, this material can have an ideal permeability selectivity for He/CH_4_ = 2.7 and for H_2_/CH_4_ = 4.5. In the case of ethane/methane and n-butane/methane, the ideal permeation selectivity is close to 1, despite the high dissolution selectivity (7.7 and 40.8, respectively). 

**Figure 5:**
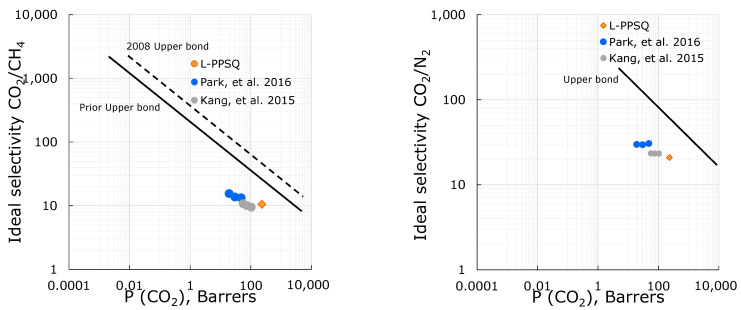
Upper bound [51] correlation for CO_2_/CH_4_ and CO_2_/N_2_ separation for L-PPSQ synthesized in this work and the closest analogs presented in the literature [49,52].

The most interesting property is that the gas permeability coefficient for carbon dioxide is relatively high. It is worth noting that the high-molecular-weight L-PPSQ obtained in this work demonstrates a more than twofold higher permeability coefficient than its cross-linked analogs [49]. This value of the CO_2_ permeability coefficient provides a higher ideal selectivity of the material for carbon dioxide: CO_2_/CH_4_ = 10.5 and CO_2_/N_2_ = 20.9. The separating properties of the material thus obtained outline the range of potential applications for CO_2_ extraction from natural and flue gases. Similar results were obtained in the early 1990s. It was shown [50] that the ideal CO_2_/CH_4_ gas vapor selectivity of L-PPSQ was about 9. This is noticeably higher than that of other polysiloxanes, such as polydimethylsiloxane, for example. However, the authors mention the low mechanical strength and high brittleness of the L-PPSQ films studied, which made it difficult and even impossible to use them as membrane-forming materials. For comparison, the correlation of CO_2_ permeability and selectivity of CO_2_/N_2_ and CO_2_/CH_4_ of the polymers synthesized in this work and the closest analogs presented in the literature on the Robson diagram [51] is shown (Figure 5).

Based on the correlation shown in Figure 5, it is clear that increasing the molecular weight (Mn = 238 kDa, Mw = 578 kDa) of the L-PPSQ leads to a significant (2–5 times) increase in the permeability of the ladder polymer while maintaining increased CO_2_ selectivity.

### 3.4. Vacuum Pervaporation

**Figure 6:**
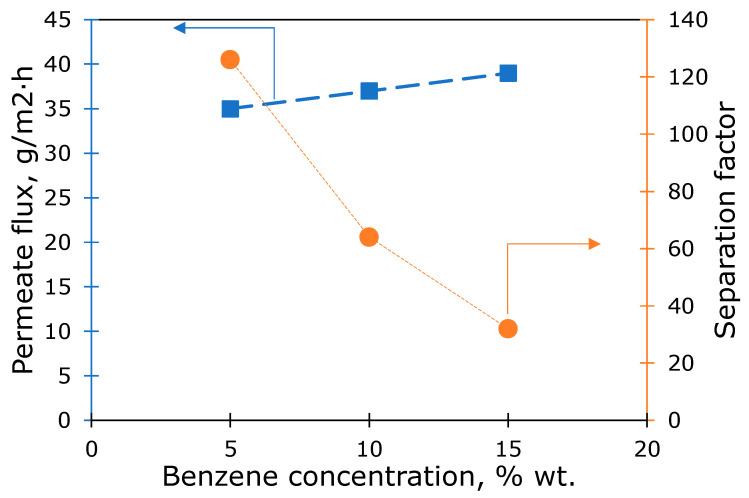
Study of the transport and separation characteristics of L-PPSQ membranes in the pervaporation separation of benzene/hexane mixtures with various compositions.

Catalytic reforming is one of the methods used to produce high-octane gasoline fractions. In this process, dehydrogenation of naphthenic hydrocarbons and dehydrocyclization of paraffines result in reformate, a product rich in aromatic hydrocarbons. Along with the advantages due to the presence of aromatic hydrocarbons in gasoline, drawbacks also exist, namely, an increase in the liability of automotive gasoline to produce carbon monoxide. In addition, benzene, whose content in gasoline should not be more than 1%, is isolated separately from aromatic compounds [53]. As an alternative to the traditional methods, i.e., extraction and hydrogenation, a membrane process—vacuum pervaporation—can be used for the separation of aromatic compounds from gasoline. In view of this, in this work, we studied L-PPSQ membranes for vacuum pervaporation in the separation of benzene-hexane mixtures. The concentration of benzene in n-hexane varied from 5 to 15 wt.%. The solution treatment temperature was 20 °C, and the pressure in the submembrane space was 0.2 mbar. Figure 6 shows the permeate flow and the separation factor as functions of the benzene concentration in the solution being processed. As one can see from Figure 6, the permeate flow increases as the benzene content in the solution increases. The maximum permeate flow over the entire range of benzene concentrations in n-hexane studied was 39 g/(m^2^·h). The partial flow of n-hexane (4.6–5.9 g/(m^2^·h)) was almost an order of magnitude smaller than that of benzene (30–33 g/(m^2^·h)) over the entire concentration range studied. The maximum value of the separation factor was 126. It should be noted that the separation factor decreases significantly (4-fold) with an increase in the concentration of benzene in hexane. This is probably due to the membrane swelling at higher benzene concentrations in the mixture, which causes a higher penetration of hexane through the membrane. A comparison of our results with the literature data showed that the L-PPSQ membranes studied in this work had the maximum benzene/hexane separation factors (Table 5).

## 4. Conclusions

In this work, a high-molecular-weight LPS was obtained, which has improved thermal and mechanical characteristics compared to literary analogs. These characteristics make it possible to obtain a polymer membrane based on it with good operational properties.

The films of high-molecular-weight L-PPSQ obtained in this work showed an improved selectivity for carbon dioxide (CO_2_/CH_4_ = 10.5; CO_2_/N_2_ = 20), which makes it a promising material for CO_2_ separation tasks. Moreover, films of high molecular weight L-PPSQ showed a record-high separation factor of benzene/n-hexane mixtures (up to 126) at permeate fluxes through the membrane comparable to well-known polymeric membranes. Thus, this membrane material is also promising for the separation of aromatic and aliphatic hydrocarbon mixtures.

## Figures and Tables

**Table 1:** Molecular mass characteristics and thermal and mechanical properties of L-PPSQ.

Mp, kDa	Mw, kDa	Mn, kDa	T_d_^5%^, °C (Air)	T_d_^5%^, °C (Argon)	E, MPa	σ, MPa	ε, %
577.9	540.4	238.1	537	587	1700	44	6

**Table 2:** Estimation of the solubility and long-range interaction of the polymer and solvents of various nature.

Compound	Solubility
L-PPSQ	−
Chloroform	+
Benzene	+
NMP	+
DMAA	+
Hexane	−
Acetone	−
Ethanol	−
Benzene/hexane 10/90	−
Benzene/hexane 30/70	Strong swelling

“+”—dissolves, “−”—insoluble.

**Table 3:** Gas transport properties of L-PPSQ.

Gas	*P*, Barrer	*D*·10^8^, cm^2^/s	*S*·10^2^, cm^3^/(cm^3^ × cmHg)
He	60	2200	0.03
H_2_	98	1000	0.10
N_2_	11	44	0.25
O_2_	33	81	0.41
CO_2_	230	60	3.8
CH_4_	22	17	1.3
C_2_H_6_	29	2.8	10
C_4_H_10_	25	0.5	53

**Table 4:** Ideal selectivities of permeability *α_P_*, diffusion *α_D_*, and dissolution *α_S_* L-PPSQ.

Gas	*α_P_*	*α_D_*	*α_S_*
He/CH_4_	2.7	129	0.02
H_2_/CH_4_	4.5	58.8	0.08
CO_2_/N_2_	20.9	1.4	15.2
O_2_/N_2_	3	1.8	1.64
CO_2_/CH_4_	10.5	3.5	2.92
C_2_H_6_/CH_4_	1.3	0.2	7.7
C_4_H_10_/CH_4_	1.1	0.03	40.8

**Table 5:** Comparison of the pervaporation results obtained with literature data.

Membrane	Conditions	Specific Permeate Flux, g·µm/(m^2^·h)	Separation Factor	Source
L-PPSQ	Benzene/hexane, 5/95%, T = 20 °C	1050	126	This study
L-PPSQ	Benzene/hexane, 10/90%, T = 20 °C	1110	64	This study
L-PPSQ	Benzene/hexane, 15/85%, T = 20 °C	1170	32	This study
3,39,4,49-diphenylsulfone-tetracarboxylic dianhydride-2,8(6)-dimethyl-3,7- diaminobenzothiophene-5,5-dioxide polyimide (DSDA-DDBT)	Benzene/hexane, 17/83%, T = 60 °C	1033	10.6	[54]
Polyurethane	Benzene/hexane, 8/92%, T = 40 °C	600	9.5	[55]
Polyurethane	Benzene/hexane, 50/50%, T = 25 °C	388	5.6	[55]
polyurethane	Benzene/hexane, 10/90%, T = 25 °C	1000	25	[56]
Poly(ethylene oxide imide)	Benzene/hexane, 40/60%, T = 60 °C	28	7.5	[54]
Poly(vinylalcohol)	Benzene/hexane, 20/80%, T = 50 °C	168	3	[57]
Polydimethylsiloxane with 6% wt. poly(oligosilsesquioxanes) *	Benzene/heptane, 0.6/99.4%, T = 70 °C	12 825	3.5	[58]

* Composite membrane.

## Data Availability

Not applicable.

## Supplementary Materials

The following supporting information can be downloaded at: https://www.mdpi.com/article/10.3390/polym15153277/s1, Figure S1: GPC curve of L-PPSQ; Figure S2: TGA curves of L-PPSQ obtained at a heating rate of 10 degrees/min in air and in argon; Figure S3: Stretching curve of L-PPSQ; Table S1: Molecular weight characteristics of L-PPSQ.
