# Supplementary material for: Pervaporation and Gas Separation Properties of High-Molecular Ladder-like Polyphenylsilsesquioxanes
Source: Polymers (Basel). 2023 Aug 2;15(15):3277. doi: 10.3390/polym15153277 (PMC10422331; doi:10.3390/polym15153277)
Supplement: Supplementary file 1 [file polymers-15-03277-s001.zip › polymers-2490834-supplementary.pdf]

Supplementary information

Pervaporation and gas separation properties of high-molecular ladder-like polyphenylsilsesquioxanes

Tatiana S. Anokhina, Tatyana O. Ershova, Anton A. Anisimov, Maxim N. Temnikov, Evgenia A. Grushevenko, Ilya L. Borisov, Alexey V. Volkov and Aziz M. Muzafarov

Table of contents

1. GPC of L-PPSQ …………………………… ……………………..…..2
2. TGA analysis…………………………………………………………......3
3. Mechanical characteristics of L-PPSQ………………………………......4

1. **GPC of ladder-like polyphenylsilsesquioxanes (L-PPSQ)**

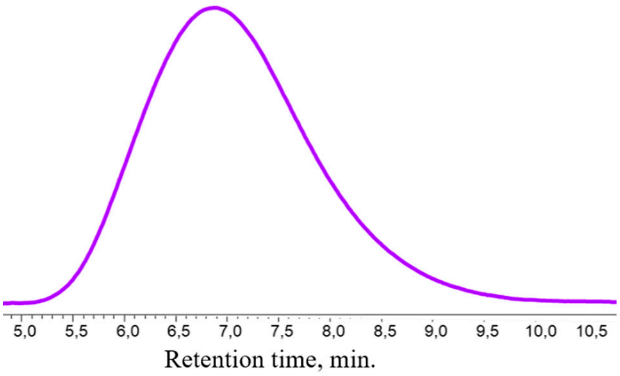

Figure S1. GPC curve of L-PPSQ.

Table S1

Molecular weight characteristics of L-PPSQ

Sample	Monomer concentration, wt%	Temperature, ⁰C	Reaction time, h	Mp, kDa	Mw, kDa	Mn, kDa	PDI
L-PPSQ	20	150	4	577,9	540,4	238,1	2,3

2. TGA analysis of L-PPSQ

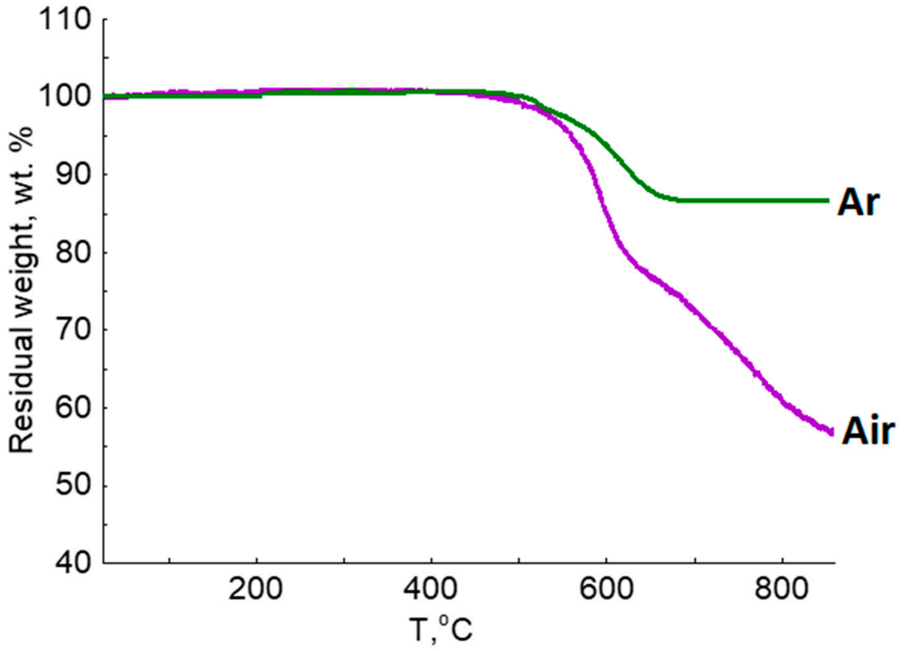

Figure S2. TGA curves of L-PPSQ obtained at a heating rate of 10 degrees/min in air and in argon.

3. **Mechanical characteristics of L-PPSQ**

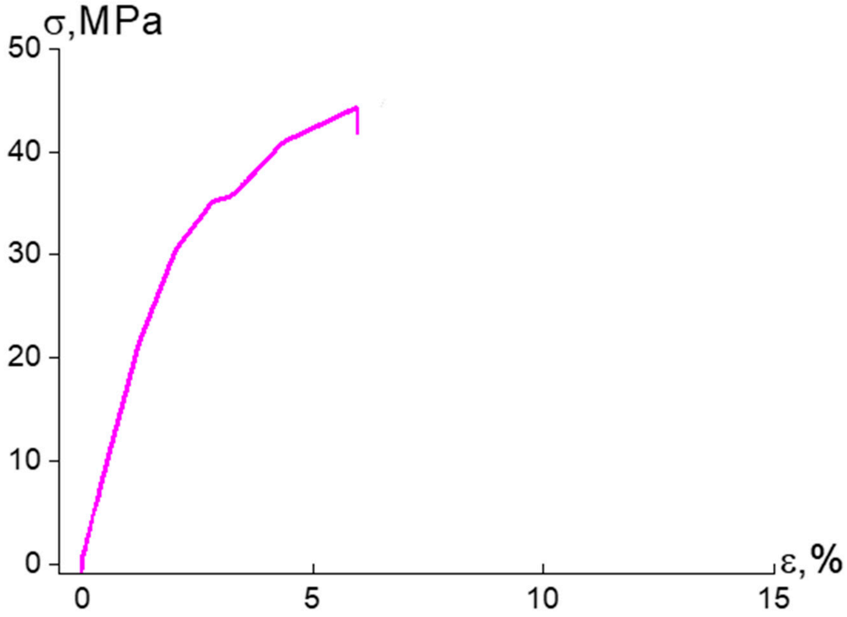

Figure S3. Stretching curve of L-PPSQ
